# Response of Pea Varieties to Damage Degree of Pea Weevil,* Bruchus pisorum* L.

**DOI:** 10.1155/2016/8053860

**Published:** 2016-03-03

**Authors:** Ivelina Mitkova Nikolova

**Affiliations:** Institute of Forage Crops, 89 General Vladimir Vazov Street, 5800 Pleven, Bulgaria

## Abstract

A study was conducted to determine the response of five pea varieties (*Pisum sativum* L.) to damage degree of* Bruchus pisorum*: Glyans, Modus, Kamerton, and Svit (Ukrainian cultivars) and Pleven 4 (Bulgarian cultivar). The seeds were classified into three types: healthy seeds (type 1), damaged seeds with parasitoid emergence hole (type 2), and damaged seeds with bruchid emergence hole (type 3) and they were sown. It was found that the weight of 1000 seeds did not affect the field germination of the pea varieties. Healthy and damaged seeds with parasitoid emergence holes (first and second seed types) provide a very good opportunity for growth and development while plants from damaged seeds with bruchid emergence holes had poor germination and vigor and low productivity. These seeds cannot provide the creation of well-garnished seeding and stable crop yields. Among tested varieties, the Ukrainian variety Glyans had considerably higher seed weight, field germination, and index germination and weak egg-laying activity of* B. pisorum* compared to others. Use of spring pea cultivars that are weakly preferred by the pea weevil in breeding programs would reduce losses due to pea weevil and provide an environmentally safer option to its control.

## 1. Introduction

The seed plays an important role in the transfer of genetic characters and improvement of qualitative and quantitative traits of production. One of the most important factors in maximizing crop yield is planting a high-quality seed. Seed size is an important physical indicator of seed quality that affects vegetative growth and is frequently related to yield, market grade factors, and harvest efficiency [1].

The effect of seed size on germination and the following seedling emergence varied widely between species and there are different hypotheses about it. Most investigators have reported a positive relationship between seedling vigor, improved stand establishment, higher productivity of crops, and greater resistance to adverse conditions during emergence of seedlings in the field with plants originating from large seeds compared to those grown from smaller seeds [2, 3]. On the other hand, Zareian et al. [4] reported that the germination rate significantly decreased by increasing seed size of wheat cultivars.

Seed germination is influenced also by many abiotic and biotic factors. Abiotic factors, such as water stress brought about by drought and salinity, limited plant germination and growth during early seedling stages [5]. On the other hand, seed beetle affects seed germination. Bruchid weevils complete their development by burrowing into and eating the vital parts of a single seed, each damaged seed yielding one adult weevil. The pea weevil is one of the most destructive pests of grain legumes [6] and cultivar improvement for bruchid resistance is among the most important strategies for mitigating the action of biotic factors.

The seed vigor is an important component that can influence crop plant density and yield [7]. That vigor is related to the germination and seed ability to grow rapidly and jointly.

Just how much damage a seed can sustain and still yield a viable seedling has not been the subject of many investigations [8]. The net effects of seed beetle infestation on the germination and recruitment of host legumes can be unpredictable [9]. If the embryo is not killed, many seeds can germinate and develop normally [10], though perhaps with fewer initial reserves. With reduced initial reserves, the seedling may be a poorer competitor than its better-provisioned counterparts. Nakai et al. [11] found that some proportions of infected seeds germinate successfully and seeds from which* Pteromalus* wasps emerged germinated more successfully than the seeds from which* Bruchus loti* adults emerged. On the other hand, Mateus et al. [12] found that the proportion of pea germinated seeds was significantly higher for nonattacked seeds from* Bruchus pisorum* compared to the attacked seeds.

In some cases, the insect clearly acts as a seed predator; larval feeding effectively kills the embryo or removes so much endosperm that the seed cannot germinate [13, 14]. Larval feeding may also create openings for pathogenic bacteria and fungi [15]. Depletion of cotyledon reserves may slow plant growth and hence reduce the probability of establishment.

Moderate beetle densities (within ranges commonly observed in nature) reduce germination frequency (probably by killing the embryo) and reduce the growth of seedlings, substantially reducing plant fitness [16]. It should not be surprising therefore that previous studies have reported highly beneficial, moderate, and highly detrimental effects of seed beetles on the fates of host seeds [17–19].

To determine the response of pea varieties to damage degree of pea weevil,* Bruchus pisorum*, we measured field germination, inhibitory effect, germination index, and productivity and its basic elements.

## 2. Material and Methods

In the experimental field of the Institute of Forage Crops, Pleven, Bulgaria (latitude: 43°25′0 N; longitude: 24°37′0 E; altitude: 230 m), during a two-year period, 2014-2015, a study was conducted to determine the response of five pea varieties (*Pisum sativum* L.) to damage degree of* Bruchus pisorum* (Coleoptera, Chrysomelidae). Pea varieties were Glyans, Modus, Kamerton, and Svit (Ukrainian cultivars) and Pleven 4 (Bulgarian cultivar). The field experiment was located in an area complying with a 2-year conversion requirement for organic production. Spring oat was the precrop. The field trial was conducted using a randomized long plot design with a sowing rate of 120 seeds germinated m^−2^, a size of harvest plot of 4 m^2^, three replications, and a natural background of soil supply with the major nutrients. In the long plot design, the replications were arranged in an elongate strip; that is, the replications are arranged one after another. The method was applied because the soil fertility was equalized. The soil type was a leached chernozem with pH_KCl_ value of 5.49 and content of total N at 34.30 mg/1000 g soil, Р_2_O_5_ at 3.72 mg/100 g soil, and K_2_O at 37.50 mg/100 g soil. No pesticides were applied.

The period of study covered years differing in meteorological conditions (Table 1). With the sum of vegetation rainfall of 277.1 mm and the average daily air temperature of 16.3°С, the year 2014 was more favorable for spring pea growth and development. The year 2015 was characterized by considerably lower sums of vegetation rainfall amounts, as, in comparison with 2014, the rainfall was lower by 38.7% and the average daily air temperature was higher by 0.8°C. In addition, the higher relative humidity in 2014 contributed to the earlier seed germination.

For every year, the seeds of pea varieties were classified into three types: healthy seeds (type 1), damaged seeds with parasitoid emergence hole (type 2), and damaged seeds with bruchid emergence hole (type 3). The three seed types were sown by taking 750 seeds (250 seeds in each replication) for every seed type.

Fifty seeds were sown by hand in a single row of the plot in 30 mm depth and 1.0 m length, 0.2 m apart, as each pea variety had three replicates for each seed type. Each plot had five rows. The number of seedlings which emerged was counted at regular intervals until an increase in the number of seedlings on two successive counts was not observed.

The inhibitory effect (IE, %) from damage by* B. pisorum* on germination was calculated by the following formula:

(1) $$\begin{array}{c} IE=\left[\;\frac{\left(a-b\right)}{a}\;\right]\times 100 \end{array}$$

(see [20]), where *a* is the number of germinated healthy seeds; *b* is the number of germinated damaged seeds from (a) the second type and (b) the third type.

The germination index (GI) for the tree types of seeds was calculated as described in the Association of Official Seed Analysts [21] by the following formula:

(2) GI=Number  of  germinated  seedsDays  of  the  first  count+⋯+Number  of  germinated  seedsDays  of  the  final  count.

The following parameters were accounted for: oviposition and productivity and its basic elements. The number of eggs per pod, which were laid by* B. pisorum*, was recorded at the flowering stage as 50 pods per plot were reviewed to count the number of eggs.

The data were averaged. They were subjected to one-way ANOVA, and the means were compared by Tukey's test at 5% probability (*P* ≤ 0.05). The Multiple Regression Analysis of Statgraphics Plus [22] for Windows Ver. 2.1 software program was used.

## 3. Results and Discussion

In the present study, varieties had significant differences in the weight of 1000 seeds in the first seed type as Kamerton had the highest weight, while Pleven 4 had the lowest weight (Table 2). Similar to the first type, in the second and third seed types, we observed a significant difference as Kamerton and Svit had the highest weight of 1000 seeds.

The weight of 1000 seeds did not affect the field germination of the plants.

In the first type of seeds (healthy seeds), significant differences in the germination between varieties were not observed (Table 3). The values of germination were high, and the seeds ensured the development of normal plants with capacity for maximum yield. Farhoudi and Motamedi[23] reported that, in safflower, there was no significant difference between seed size and germination. Also, in barley, germination was in the range 97.5%–98.5% in four groups of seed sizes and there was no significant difference between them [1]. These results are in agreement with our findings.

The second type of seeds was characterized by an average of 19.9 percent lower germination to the first type. A significant difference was found only between Glyans and Svit. A significant difference was found between Pleven 4 and Glyans in the third seed type as Pleven 4 had a lower percent of germination.

In a comparative analysis of the three seed types, damaged seeds with bruchid emergence holes (third type of seeds) were characterized by less established field germination compared to the first and second types (Table 3). Differences were significant (in Svit, there was a significant difference between the first and third seed types). The field germination decreased on average by 67.4% compared to the first seed type and with 59.4% compared to the second type. Probably, the lower content of nutrients failed to compensate for the larvae damage and seed cannot germinate.

Traits as a weight of 1000 seeds and field germination at first and second seed types mainly had similar values. This is probably due to early mortality of larvae of the weevils, before they were consuming considerable amounts of seed nutrients. Therefore, the embryonic tissue was less damaged in the second type and there was no statistically significant reduction in the field germination compared to the germination of the healthy seeds. Similar results were reported by Mateus et al. [12], who did not find significant differences in germination and vigor in a comparative analysis of the first and second types of seeds.

According to some authors [11, 24], if beetle damage acts as a scarifying agent, it can have a large, positive effect on the frequency of germination. Takakura [19] concluded that beetle infestation was considered a prerequisite to successful germination. Nakai et al. [11] reported that parasitoid wasps, which attacked seed beetle larvae, may accomplish scarification but do not consume enough tissue to kill seeds.

In the present study, parasitized weevil larvae by the parasite* Triaspis thoracica* Curtis probably died before consuming considerable amounts of nutrients from the seeds.

Unlike the first and second types of seeds, larvae at the third type of damaged seeds, feeding effectively, killed the embryo or destroyed much of the endosperm, as a result of which the seeds cannot germinate or the germination rate was strongly reduced. Similar results were reported by other authors [13, 25, 26]. No parasitized larvae consumed foods with much higher intensity in comparison to the parasitized ones [11], which results in low germination and high inhibiting effect.

A similar trend was found with respect to the germination index (GI) as the germination of the first seed type exceeded the second by an average of 26.8%. There were statistical differences except for the Bulgarian variety. The lowest GI characterized damaged seeds with bruchid emergence holes. The average values of decrease to the first and second seed types were 74.2 and 64.7 percent, respectively. A statistically significant difference between the three types of seeds was found in Ukrainian varieties. In Pleven 4, a significant difference was observed between the first and second seed types and third type.

The inhibitory effect (IE, %) from* B. pisorum* damage on field germination at the second seed type was less pronounced with slight differences between varieties. IE_1_ was on average 23.4%. Only Svit had a significantly higher value compared to Kamerton and Pleven 4. In the third seed type, IE_2_ was on average 68.3%, and it was three times higher in comparison to the second type. Differences between varieties were not established, but there was a significant difference between IE_1_ and IE_2_.

Significant differences between the types of seeds were established between Glyans and Pleven 4 as the Ukrainian variety had a higher seed weight (at the three types of seeds), field germination (at the third type), and index germination (at the first seed type).

With regard to the egg-laying activity of pea weevil females, no significant differences were found in the oviposition on the plant varieties from the three types of seeds (Table 4). An exception was observed only in Pleven 4, where the preference of the pea weevil to lay on plant pods from the third type of seeds was lower, and the differences were significant (between the first and third and second and third types of seeds). There was a tendency to reduce egg-laying activity from the first to the third type of seeds, which probably was related to the selection of weevils to lay their eggs on more well-developed pods.

In a comparative analysis of varieties, certain differences were established with respect to the types of seeds and the laying activity of* B. pisorum*. Glyans was the least preferred, followed by Modus. The proportion of infected pods and the number of eggs on plants from the first and second seed types in Glyans were significantly lower compared to these in Kamerton and Pleven 4 (except for the number of eggs on plants from the first seed type in Kamerton).

A genetic potential of varieties and environmental factors determined variety productivity and the accompanying elements [27]. Among plants from the first seed type, the Bulgarian variety compared to the Ukrainian one had the greatest plant height and number of pods and seeds (differences between Pleven 4 and Ukrainian varieties were significant), but lower productivity per plant (Table 5). Plants from three seed types in Svit and Kamerton had significantly higher productivity compared to Pleven 4 (except for plants from the third type in Kamerton). Their productivity exceeded other Ukrainian varieties, but significant differences were not found.

A similar trend was found with respect to the productivity and its basic elements in plants from the second seed type as Svit and Kamerton had higher productivity than others. Significant differences in the productivity of plants from the first and second seed type were not found.

The damage from pea weevil in the third seed type was related to the development of plants with reduced vigor and height (average of 15.8%) and number of pods (average of 21.4%) and seeds (average of 24.2%). Differences compared to normally developing plants were significant. The most pronounced decrease in the height, number of pods and seeds, and productivity of plants from the third seed type compared to the first type was found in Pleven 4. Glyans, followed by Svit, was characterized with the lowest reduction. One of the reasons was probably related to the amount of reserve nutrients accumulated in seeds and their potential. The depletion of the reserves of the cotyledon at the third type of seeds slowed down plant growth, reduced the probability of recovery, and resulted in highly dwarfed growth (height) and productivity of plants.

Sabbour and Abd-El-Aziz [28] indicated that bruchid beetles attacking legume seeds caused severe damage to the quality and quantity of the crop. The total yield of a heavily infested pea crop by* B. pisorum* may be reduced by more than 85% in Ethiopia [29]. Even with only a small amount of actual biological losses, economic losses can reach up to 100% [30]. The seeds of legumes, once damaged by storage insects, are no longer fit for planting (due to poor germination) [25, 31].

In the present study, in addition, the size of the pods was measured (length and width) and results showed that pea weevil damage did not affect them (Table 5).

In general, healthy and damaged seeds with parasitoid emergence holes (first and second seed types) provide a very good opportunity for growth and development while plants from damaged seeds with bruchid emergence holes had poor germination and vigor and low productivity. These seeds cannot provide the creation of well-garnished seeding and stable crop yields.

The results of the analysis in Table 6 showed that the linear component in the regression of productivity related to the investigated productivity traits was significant only for the third seed type. Among productivity traits, the highest influence on pea productivity had the number of pods of the plant varieties from the first, second, and third types of seeds (0.356, 0.806, and 1.630, resp.) as a significant difference was found only for the third seed type. The impact of the plant height on the productivity was low. Use of spring pea cultivars that are weakly preferred by the pea weevil in breeding programs (e.g., Kamerton) would reduce losses due to pea weevil and provide an environmentally safer option to* B. pisorum* control.

## 4. Conclusions

Healthy and damaged seeds with parasitoid emergence holes (first and second seed types) provide a very good opportunity for growth and development while plants from damaged seeds with bruchid emergence holes had poor germination and vigor and low productivity. These seeds cannot provide the creation of well-garnished seeding and stable crop yields.

Among tested varieties, the Ukrainian variety Glyans had considerably higher seed weight, field germination, and index germination and weak egg-laying activity of* Bruchus pisorum* compared to others.

## Figures and Tables

**Table 1:** Weather data in the Pleven region.

Month	Ten-day periods	Temperature (°C)			Relative humidity (%)			Rainfall (mm)		
		2014	2015	Average	2014	2015	Average	2014	2015	Average
April	Min.	10.0	9.4	9.7	62.2	26.1	44.2	139.8	43.6	91.7
	Max.	15.9	16.3	16.1	84.5	45.6	65.1			
	Average	**12.3**	**12.2**	**12.3**	**76.3**	**37.8**	**57.1**			

May	Min.	15.0	12.3	13.7	51.9	50.2	51.1	83.0	30.6	56.8
	Max.	21.3	25.3	23.3	78.9	74.4	76.7			
	Average	**16.7**	**18.8**	**17.8**	**70.0**	**65.9**	**68.0**			

June	Min.	18.0	13.3	15.7	48.9	44.8	46.9	54.3	95.7	75.0
	Max.	24.3	27.3	25.8	74.3	75.4	74.9			
	Average	**19.9**	**20.3**	**20.1**	**64.8**	**64.0**	**64.4**			

**Table 2:** Weight of 1000 seeds (g) of seed types in pea cultivars.

Seeds	Glyans	Svit	Kamerton	Modus	Pleven 4	LSD_0.05%_
Type 1	212.92 b^1^/a^2^	222.35 c/a	251.75 d/b	226.32 c/c	137.16 a/b	6.922
Type 2	209.78 b/a	213.60 b/a	247.74 c/b	213.59 b/b	131.91 a/ab	6.979
Type 3	209.07 c/a	223.09 d/a	223.29 d/a	199.65 b/a	134.61 a/a	7.889
LSD_0.05%_	11.579	9.544	9.736	7.756	5.069	

^1^Means in each row followed by the same letters are not significantly different (*P* > 0.05).

^2^Means in each column followed by the same letters are not significantly different (*P* > 0.05).

**Table 3:** Seed damage ratings by *Bruchus pisorum* in pea cultivars.

Traits	Glyans	Svit	Kamerton	Modus	Pleven 4	LSD_0.05%_
FG, type 1	78.3 b^1^/c^2^	64.3 ab/b	62.0 ab/b	71.5 ab/b	53.0 ab/b	23.242
FG, type 2	61.0 b/b	46.8 a/ab	49.5 ab/b	53.0 ab/b	52.5 аb/b	14.126
FG, type 3	25.5 b/a	20.5 ab/a	21.3 ab/a	23.0 ab/a	16.8 a/a	8.672
LSD_0.05%_	12.136	27.445	20.025	19.812	19.672	
GI, type 1	84.7 c/c	59.5 b/c	59.7 b/c	74.8 c/c	40.4 a/b	10.861
GI, type 2	59.7 c/b	33.5 a/b	44.7 b/b	45.2 b/b	50.5 bc/b	10.771
GI, type 3	20.3 a/a	13.2 a/a	14.1 a/a	18.0 a/a	16.8 a/a	8.600
LSD_0.05%_	14.473	12.478	11.043	13.649	10.678	
IE_1_	21.8 ab/a	29.6 b/a	20.7 a/a	25.8 ab/a	19.2 a/a	8.690
IE_2_	67.0 a/b	69.7 a/b	66.1 a/b	67.7 a/b	70.9 a/b	9.341
LSD_0.05%_	16.007	14.936	14.717	14.985	14.819	

FG: field germination, %; GI: germination index, %; IE_1_, %: inhibitory effect from *B. pisorum* damage on field germination at the second seed type; IE_2_, %: inhibitory effect from *B. pisorum* damage on field germination at the third seed type.

^1^Means in each row followed by the same letters are not significantly different (*P* > 0.05).

^2^Means in each column followed by the same letters are not significantly different (*P* > 0.05).

**Table 4:** Laying activity of *Bruchus pisorum* in pea cultivars.

Variety	*A*				*B*			
	Type 1	Type 2	Type 3	LSD_0.05%_	Type 1	Type 2	Type 3	LSD_0.05%_
Glyans	43.59 a^1^/a^2^	32.50 a/a	48.27 b/a	18.541	0.61 a/a	0.45 a/a	0.55 bc/a	0.483
Svit	43.86 a/a	52.78 b/a	48.00 b/a	13.368	0.89 ab/a	0.72 ab/a	0.72 cd/a	0.615
Kamerton	57.77 b/a	54.29 b/a	42.86 ab/a	15.164	1.00 ab/a	0.91 b/a	0.50 ab/a	3.730
Modus	46.67 a/a	39.29 a/a	43.75 ab/a	14.413	0.67 ab/a	0.68 ab/a	0.79 d/a	0.414
Pleven 4	64.71 b/b	60.50 b/b	37.40 a/a	13.340	1.10 b/b	1.00 b/b	0.33 a/a	0.450
Average	**12.94**	**12.10**	**7.48**		**0.85**	**0.75**	**0.58**	
LSD_0.05%_	10.959	13.24	9.505		0.446	0.341	0.215	

*A*: proportion of infected pods in plants from the three seed types, %.

*B*: number of eggs per pod from the three seed types.

^1^Means in each column followed by the same letters are not significantly different (*P* > 0.05).

^2^Means in each row followed by the same letters are not significantly different (*P* > 0.05).

**Table 5:** Productivity and its basic elements in pea cultivars.

Traits	Glyans	Svit	Kamerton	Modus	Pleven 4	LSD_0.05%_
*H*, type 1	52.52 a^1^/b^2^	53.98 a/b	66.79 b/b	54.11 a/b	81.88 c/b	2.812
*H*, type 2	50.10 a/ab	51.50 a/ab	63.45 b/b	51.25 a/ab	76.40 c/b	6.681
*H*, type 3	46.30 a/a	46.95 a/a	55.42 b/a	46.04 a/a	65.62 c/a	5.982
LSD_0.05%_	5.432	6.085	6.498	5.721	9.174	
NP, type 1	4.57 a/a	5.47 b/b	4.79 a/b	4.40 a/b	5.44 b/c	0.486
NP, type 2	4.22 ab/a	5.04 c/ab	4.13 ab/ab	4.00 a/ab	4.75 bc/b	0.649
NP, type 3	3.85 a/a	4.55 b/a	3.47 a/a	3.63 a/a	3.89 a/a	0.505
LSD_0.05%_	0.713	0.632	0.725	0.672	0.668	
NS, type 1	17.25 a/c	18.62 b/b	18.42 b/a	17.84 ab/c	24.62 c/c	1.014
NS, type 2	15.47 a/b	17.32 b/b	14.94 a/a	15.52 a/b	21.23 c/b	1.639
NS, type 3	13.47 a/a	14.89 ab/a	14.00 a/a	13.14 a/a	17.82 b/a	3.371
LSD_0.05%_	1.642	1.342	5.285	1.018	2.25	
*P*, type 1	3.59 ab/b	4.01 b/b	4.02 b/b	3.90 ab/b	3.37 a/b	0.548
*P*, type 2	3.38 ab/ab	3.72 b/ab	3.68 b/b	3.34 ab/ab	2.86 a/ab	0.548
*P*, type 3	2.73 ab/a	3.13 b/a	2.75 ab/a	2.54 ab/a	2.30 a/a	0.662
LSD_0.05%_	0.723	0.611	0.835	1.056	0.777	
LP, type 1	5.41 ab/b	5.63 ab/a	5.71 b/b	5.37 ab/a	5.13 a/a	0.522
LP, type 2	4.98 ab/ab	5.40 b/a	5.06 ab/a	5.02 ab/a	4.64 a/a	0.606
LP, type 3	4.85 ab/a	5.20 b/a	4.71 ab/a	4.82 ab/a	4.40 a/a	0.513
LSD_0.05%_	0.43	0.857	0.573	0.676	0.773	
WP, type 1	0.69 a/a	0.79 a/a	0.83 a/a	0.85 a/a	0.69 a/a	0.398
WP, type 2	0.67 a/a	0.77 a/a	0.79 a/a	0.77 a/a	0.67 a/a	0.382
WP, type 3	0.63 a/a	0.74 a/a	0.75 a/a	0.75 a/a	0.64 a/a	0.307
LSD_0.05%_	0.922	0.292	0.227	0.156	0.09	

*H*: height, cm; NP: number of pods/1 plant; NS: number of seeds/1 plant; *P*: productivity, g/1 plant; LP: length of 1 pod, cm; WP: width of 1 pod, cm.

^1^Means in each row followed by the same letters are not significantly different (*P* > 0.05).

^2^Means in each column followed by the same letters are not significantly different (*P* > 0.05).

**Table:** (a) Healthy seeds (type 1)

ANOVA	df		SS	MS	*F*		Significance *F*	
Regression	3.000		0.220	0.073	0.713		0.678	
Residual	1.000		0.103	0.103				
Total	4.000		0.323					

Productivity	Coefficients	Standard error	*t* stat.	*P* value	Lower 95%	Upper 95%	Lower 95.0%	Upper 95.0%

Intercept	4.273	1.605	2.663	0.229	−16.114	24.661	−16.114	24.661
*H*	0.018	0.034	0.545	0.682	−0.409	0.446	−0.409	0.446
NP	0.356	0.466	0.764	0.585	−5.569	6.281	−5.569	6.281
NS	0.175	0.164	1.065	0.480	−2.264	1.913	−2.264	1.913

**Table:** (b) Damaged seeds with parasitoid emergence hole (type 2)

ANOVA	df		SS	MS	*F*		Significance *F*	
Regression	3.000		0.469	0.156	18.341		0.170	
Residual	1.000		0.009	0.009				
Total	4.000		0.477					

Productivity	Coefficients	Standard error	*t* stat.	*P* value	Lower 95%	Upper 95%	Lower 95.0%	Upper 95.0%

Intercept	3.062	0.519	5.898	0.107	−3.535	9.658	−3.535	9.658
*H*	0.013	0.007	1.776	0.326	−0.078	0.103	−0.078	0.103
NP	0.806	0.167	4.835	0.130	−1.312	2.923	−1.312	2.923
NS	0.235	0.042	5.658	0.111	−0.763	0.293	−0.763	0.293

**Table:** (c) Damaged seeds with bruchid emergence hole (type 3)

ANOVA	df		SS	MS	*F*		Significance *F*	
Regression	3.000		0.373	0.124	6538.771		0.009	
Residual	1.000		0.000	0.000				
Total	4.000		0.373					

Productivity	Coefficients	Standard error	*t* stat.	*P* value	Lower 95%	Upper 95%	Lower 95.0%	Upper 95.0%

Intercept	0.616	0.044	14.103	0.045	−1.171	−0.061	−1.171	−0.061
*H*	0.103	0.001	70.255	0.009	0.084	0.122	0.084	0.122
NP	1.630	0.016	102.506	0.006	1.428	1.832	1.428	1.832
NS	0.571	0.007	84.574	0.008	−0.657	−0.485	−0.657	−0.485

*H*: height, cm; NP: number of pods; NS: number of seeds.
